# Validity and reproducibility of arterial pulse wave velocity measurement using new device with oscillometric technique: A pilot study

**DOI:** 10.1186/1475-925X-4-49

**Published:** 2005-08-23

**Authors:** Madireddy Umamaheshwar Rao Naidu, Budda Muralidhar Reddy, Sridhar Yashmaina, Amar Narayana Patnaik, Pingali Usha Rani

**Affiliations:** 1 Department of Clinical Pharmacology & Therapeutics, Nizam's Institute of Medical Sciences, Punjagutta, Hyderabad, 500082, India; 2 Department of Cardiology, Nizam's Institute of Medical Sciences, Punjagutta, Hyderabad, 500082, India

**Keywords:** Pulse wave velocity, Arterial stiffness index, Validity, Oscillometric device

## Abstract

**Background:**

Availability of a range of techniques and devices allow measurement of many variables related to the stiffness of large or medium sized arteries. There is good evidence that, pulse wave velocity is a relatively simple measurement and is a good indicator of changes in arterial properties. The pulse wave velocity calculated from pulse wave recording by other methods like doppler or tonometry is tedious, time-consuming and above all their reproducibility depends on the operator skills. It requires intensive resource involvement. For epidemiological studies these methods are not suitable. The aim of our study was to clinically evaluate the validity and reproducibility of a new automatic device for measurement of pulse wave velocity that can be used in such studies.

**Methods:**

In 44 subjects including normal healthy control and patients with coronary artery disease, heart brachial, heart ankle, brachial ankle and carotid femoral pulse wave velocities were recorded by using a new oscillometric device. Lead I and II electrocardiogram and pressure curves were simultaneously recorded. Two observers recorded the pulse wave velocity for validation and one observer recorded the velocity on two occasions for reproducibility.

**Results and Discussion:**

Pulse wave velocity and arterial stiffness index were recorded in 24 control and 20 coronary artery disease patients. All the velocities were significantly high in coronary artery disease patients. There was highly significant correlation between the values noted by the two observers with low standard deviation. The Pearson's correlation coefficient for various velocities ranged from (r = 0.88–0.90) with (p < 0.0001). The reproducibility was also very good as shown by Bland-Altman plot; most of the values were lying within 2 SD. The interperiod measurements of pulse wave velocity were also significantly correlated (r = 0.71 – 0.98) (P < 0.0001). Carotid-femoral pulse wave velocity was found to correlate significantly with heart brachial, heart ankle, brachial ankle pulse wave velocity and arterial stiffness index values. Reproducibility of our method was good with very low variability in both interobserver and interperiod analysis.

**Conclusion:**

The new device "PeriScope" based on oscillometric technique has been found to be a simple, non-invasive and reproducible device for the assessment of pulse wave velocity and can be used to determine arterial stiffness in large population based studies.

## Background

Interest in, and measurement of the velocity of arterial wave propagation as an index of vascular stiffness and vascular health dates back to the early part of the last century. Many methodologies; both invasive and non-invasive have been applied to the assessment of arterial elasticity in vivo. The assessment of cardiovascular risk is one of the most important tasks. The predictive value of pulse wave velocity (PWV) is becoming increasingly recognized and it is one of the classical indices of arterial stiffness (AS). Several arterial assessments improve risk stratification. Three of the most cost-effective assessment parameters are pulse pressure, arterial stiffness and ankle brachial index. Arterial stiffness can be directly measured by non-invasive techniques like computerized oscillometry, tonometry and ultrasonography [1].

In one study [1] of 30 patients with and without cardiovascular disease (CVD), the diagnostic accuracies of these techniques were as follows: arterial stiffness 85%, pulse pressure 71% and endothelial function 58%. Pulse pressure is independently related to all cause mortality, but only marginally related to cardiovascular mortality, indicating that specific assessment of AS with PWV, may be of greater value in the evaluation of risk [1]. PWV measurements by ultrasonography are more time consuming, requires substantial training and dedicated staff.

A recent important observation, that AS is an independent predictor of cardiovascular mortality [2-4], has gained greater interest. Increased stiffness may precede the onset of clinically overt atheromatous disease [5]. Early identification of individuals at risk, by improved detection of changes in stiffness may help in providing beneficial intervention at an early stage [6]. Relevance and implications of possible therapy of arterial stiffness have led to wide spread interest in its measurement, resulting in large number of availability of commercial devices [7]. There is no gold standard to assess AS [8].

Recently non-invasive methods to measure AS have become available and are relatively easy to perform [9-12]. A simple device to measure PWV has been developed which measures brachial ankle pulse wave velocity (ba PWV) using an oscillometric method [13]. As it measures peripheral artery velocity, unlike aortic PWV the clinical significance may differ. Aortic PWV >13 m/s is a particularly strong predictor of cardiovascular mortality in hypertension [14]. Although ba PWV measurement is simple and non-invasive, the sensitivity and specificity in predicting coronary artery disease were only 62% and 29% [15]. A more accurate and integrated information of AS may therefore be obtained by using a combination of techniques based on different models [7]. There is a need to quantify the extent to which measures of AS can improve risk stratification and to determine whether its reduction is capable of predicting clinical benefit. In the present study, we have assessed a newly developed simple non-invasive device, which measures both peripheral and aortic PWV using an oscillometric method.

## Methods

Total twenty-four male healthy subjects with mean age 24.7 ± 5.5 years, mean height 168.3 ± 5 cm and mean weight 52.6 ± 6.4 kgs were recruited for interperiod and interobserver reproducibility study. All the subjects gave their consent for the study, approved by the Institutional Ethics Committee, Nizam's Institute of Medical Sciences, Hyderabad. Before the inclusion in the study, subjects were thoroughly examined clinically to confirm the inclusion criteria. No subject was on any medication and had normal blood pressure, CVS, renal, hepatic functions, blood sugar, serum cholesterol and uric acid levels.

All subjects refrained from smoking and caffeine containing beverages at least 24 hours before the measurements. The entire test was performed in the morning, after 10 hours over night fasting condition in a quite room with controlled temperature. In each subject two sequences of measurements were performed, and their mean was considered for analysis. All procedures were repeated by two observers (observer 1 and observer 2) for analysis of the inter-observer reproducibility, between the two sequences of measurements of PWV, the BP cuffs were rewrapped at each measurement. For inter period reproducibility assessment, PWV was measured twice by the same observer with an interval of at least one day between the two measurements.

Additionally 20 patients (9 male & 11 female) with CAD having mean age 50 ± 13 years, mean height 157.9 ± 10 cm, mean weight 67 ± 16 kgs were also included in interobserver study from the OPD. History of diabetes mellitus, hypertension, smoking, and drug use were recorded. All patients had coronary angiographically confirmed CAD. Patients were allowed to take their prescribed medication during the study period. All patients gave their consent for study participation. Every time the PWV recording was carried out at least 10 minutes after resting.

The subjects were examined in supine position. Electrodes of electrocardiogram were placed on ventral surface of both wrists and medial side of ankles and BP cuffs were wrapped on both upper arm brachial artery and above tibial artery of ankles. The cuffs were connected to a plethysmographic sensor that determines volume pulse form and an oscillometric pressure sensor that measures blood pressure volume waveforms from the brachial and tibial arteries. All the pressure recordings were done for about 10 seconds and data was stored in the computer for analysis.

New device (PeriScope, developed by Genesis Medical Systems, Hyderabad, India) is an 8-channel real-time PC based simultaneous acquisition and analysis system. The acquisition rate is 200 samples/second, which is sufficient because the significant frequency content of the pressure as well as ECG waveform is not more than 40 Hz. According to Nyquist's criteria the minimum sampling rate should be 80 samples/second. Hence a sampling rate of 200 Hz per second is optimum. It supports a sophisticated digital signal-processing algorithm to calculate all the results. System has dedicated hardware module connected to 4 ECG electrodes and 4 Blood pressure measuring cuffs. It is very user friendly and fully automatic. Once started, the test recording completes itself by displaying results directly. The report contains 8-second traces of Lead I and II ECG, all Pressure Pulse Waveforms and all calculated results. Device has a built-in database that can be used to store patient folders for further referrals at any point of time. PeriScope is a PC based low cost instrument. When used with a laptop, it can be carried to remote locations. It uses of ECG as marker. It does not use phonocardiogram. PeriScope thus facilitates use in epidemiological studies.

### Calculation of pulse wave velocity by oscillometric method

This method measures the blood pressure by detecting the pulsation of the artery, which is caused by the heart, as the pressure oscillation in the cuff. When the cuff around< the upper arm is fully inflated, blood flow stops but pulsation of the artery continues and causes oscillation of the pressure in the cuff. As the pressure in the cuff is decreased slowly, the amplitude of the pressure oscillation in the cuff gradually increases and eventually reaches to a peak. Further decrease of the cuff pressure causes the oscillation amplitude to decrease. Cuff pressure when the oscillation reaches a peak, is taken as the mean arterial pressure (MAP).

**Figure 1:**
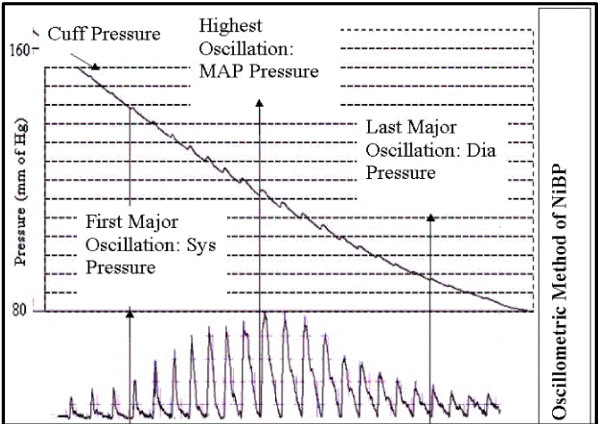
**Computerized oscillomertry**. Atypical computerized Oscillometric pattern of pressure.

Figure 1 shows the oscillometric pressure pattern. As this maximum amplitude oscillation in each limb is detected, the pulse waveform along with ECG Lead I and II are stored simultaneously in the PC memory. These waveforms are used to detect various pulse wave velocities as described below.

Pulse wave velocity is the speed at which the blood pressure pulse travels from the heart to the peripheral artery after blood rushes out during contraction. It is mainly used to evaluate stiffness of the artery wall. Pulse wave velocity increases with stiffness of the arteries.

With new device, the PTT (Pulse Transit Time) of each segment is calculated from the waveform taken from each sensor.



It calculates heart-brachial PWV of both upper limbs, heart-ankle PWV of both lower limbs, brachial-ankle PWV of both right and left limb pairs and effective estimated carotid-femoral PWV is calculated.







Where Lha = Distance between heart and respective ankle.

Lhb = Distance between heart and respective brachium.

Lba = Distance between respective brachium and ankle.

Distances were measured by direct superficial measurement with a measuring tape of 1 mm resolution as follows:

Heart to brachial distance = Heart to shoulder + shoulder to midpoint of brachial cuff.

Heart to ankle distance = Heart to midpoint of ankle cuff.

Brachial ankle distance = Heart to brachial distance + Heart to ankle distance

**Figure 2:**
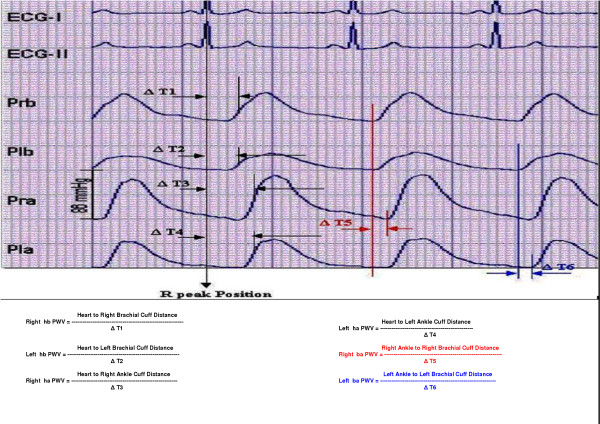
**Pulse Wave Velocity calculations**. Reference points of ECG and four pressure waveforms consider for calculation of pulse transit time and PWV.

Pulse Transit Time (PTT) between heart and extremity (i.e. PTT hb and PTT ha) is calculated using R wave as pulse start maker and maximum pressure gradient as pressure wave arrival marker. This is due to the fact that the QRS complex designates highest-pressure deviation in the left ventricle where as maximum pressure gradient marks the highest-pressure deviation in the artery of the extremity (Fig. 2). A proprietary software algorithm is used to analyze the ECG and pressure waveforms. From this analysis the pre-ejection period is calculated and deducted from each "R-wave to max. pressure gradient" time. This gives the actual pulse transit time.

Pulse Transit Time (PTT) between brachium and respective ankle is calculated as the time difference between the feet of respective pulse wave originated by the same QRS complex. The carotid femoral PWV (C-F PWV) is estimated from the composite brachial ankle pulse wave velocity (ba PWV) found out by averaging left and right ba PWV. The regression analysis between ba PWV and C-F PWV yields the following equation:

Estimated carotid femoral PWV = 0.8333 * (Avg. ba PWV) - 233.33.

This equation is arrived at by internal data collection and confirmed by studies conducted elsewhere [13].

### Derivation of oscillometric envelopes

Oscillometric envelope is a graphical depiction of compressibility of the artery. It is derived from the oscillations in the artery when the BP cuffs are deflating while taking the BP reading. It is the graph of amplitude of oscillations verses the instantaneous pressure in the blood pressure cuff. In a normal arterial condition, the shape of the oscillometric envelope is like a bell, when arteries are stiffened or atherosclerosed the oscillometric envelope flattens out.

### Calculation of arterial stiffness index

**Figure 3:**
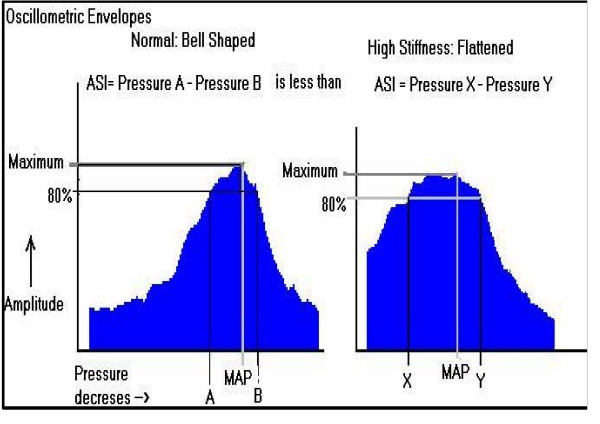
Oscillometric Envelopes.

Arterial stiffness index is another measure of arterial stiffness. It quantifies the shape of the oscillometric envelope. As the arterial stiffness increases, it becomes harder to collapse the arteries by applying external pressure; hence the oscillometric envelope becomes flatter as the stiffness increases. The ASI value gives a clear indication of this flattening process (Fig. 3). The higher the stiffness, the higher the ASI values. It is calculated as:

ASI = (Systolic side Value of cuff pressure at 80% of maximal oscillation amplitude of cuff) - (Diastolic side Value of cuff pressure at 80% of maximal oscillation amplitude of cuff).

The device acquires and computes the following parameters simultaneously: 2 channels of ECG, brachial BP of both limbs, ankle BP of both limbs, 4 channels of pulse pressure waveforms, mean arterial pressure (MAP), % MAP, ABI, pulse wave velocities, UT (Upstroke Time), arterial stiffness index.

### Statistics

Data are expressed as mean ± SD. Pearson's correlation analysis, determination of coefficient of variation and Bland-Altman plotting were performed for the assessment of validity and reproducibility [21]. When two series of paired measurements were compared, the results were analyzed in two steps according to the recommendations of Bland and Altman. First, the correlation between measurement values (equation of the linear relationship, correlation coefficient r, and P value) was investigated. The first step was used to gauge the degree of agreement between two series of measurements. Second, the relative (positive or negative) differences between each pair of measures were plotted against the mean of the pair to make sure that no obvious relation appeared between the estimated value mean and difference. The mean difference and the SD of the differences estimated the lack of agreement between the two measurements. Unpaired student 't' test was used for comparisons among the two groups. Values of p < 0.05 was considered to indicate statistical significance. All the statistical analysis was performed using the Graph pad PRISM software version 4 (Graph pad software Inc. San Diego, California, USA).

## Results

Total 44 subjects (24) healthy controls and (20) CAD patients participated in the present study. The demographic characteristic of all subjects is shown in the table-1. As our aim was to study the reproducibility and validity of a new method, we have selected both healthy controls and CAD patients. The control group had lower mean age than the CAD group; similarly the BMI was also high in CAD group. As compared to control group, all haemodynamic parameters HR, SBP, DBP, PP and MAP were also significantly higher in CAD group.

**Table 1:** Demographic characteristics of healthy subjects and patients with coronary artery disease (CAD) studied.

	**All Subjects**	**Healthy subjects**	**CAD patients**
Number	44	24	20
Sex (male/female)	33/11	24/0	9/11
Age (yrs)	37.09 ± 16.69	24.67 ± 4.65	52.00 ± 13.22***
Weight (Kgs)	59.18 ± 13.73	52.67 ± 6.46	67.00 ± 16.04^##^
Height (cms)	163.5 ± 9.342	168.3 ± 5.18	157.9 ± 10.14***
BMI (Kg/m^2^)	22.26 ± 5.331	18.65 ± 2.46	26.59 ± 4.54***
Heart Rate (bpm)	71.13 ± 15.69	62.33 ± 7.56	81.80 ± 16.47***
Systolic BP (mmHg)	106.4 ± 13.46	110.3 ± 8.8	131.9 ± 21.95*
Diastolic BP (mmHg)	58.85 ± 12.99	63.08 ± 6.6	79.15 ± 11.38**
MAP (mmHg)	82.78 ± 9.68	85.1 ± 6.64	100.9 ± 15.8^#^
Pulse Pressure	46.64 ± 11.77	47.17 ± 5.59	52.7 ± 16.08***

Values are expressed as Mean ± SD.

*** p < 0.0001, ** p < 0.0003, * p < 0.01, ^# #^p < 0.0002 and ^# ^p < 0.02 Vs Healthy subjects.

The mean PWV obtained in two groups is shown in table 2. In CAD group hb PWV, ha PWV, ba PWV and C-F PWV were found to be significantly higher than control. The mean ankle ASI was 60.6 ± 16.7 in CAD group and 48.2 ± 7.6 in control. This difference in ankle ASI was significant (p < 0.001).

**Table 2:** Pulse Wave Velocity and Arterial Stiffness Index.

**Pulse Wave Velocity**	**All Subjects**	**Healthy subjects**	**CAD patients**
Number of observations	88	48	40
Heart Brachial PWV			
Mean	279.0	262.4	298.9***
SD	45.10	17.03	58.67
SE	4.808	2.459	9.276
Heart Ankle PWV			
Mean	482.8	425.1	551.9***
SD	85.97	17.28	84.50
SE	9.165	2.494	13.36
Brachial Ankle PWV			
Mean	1392	1115	1725***
SD	397.3	103.8	362.1
SE	42.36	14.99	57.25
Carotid Femoral PWV			
Mean	930.7	702.7	1204***
SD	329.2	90.54	301.8
SE	35.09	13.07	47.72
Brachial ASI			
Mean	38.08	37.41	38.89
SD	10.23	4.373	14.46
SE	1.090	0.6312	2.287
Ankle ASI			
Mean	53.84	48.22	60.59***
SD	13.97	7.601	16.73
SE	1.489	1.097	2.646

*** p < 0.0001 Vs Healthy subjects

The mean velocities obtained by the two separate observers in control and CAD group are shown in tables 3, 4 and 5. Values of all parameters obtained by two observers were found to be highly correlated with significant Pearson's correlation coefficients.

**Table 3:** Means and correlational analysis (Pearson r value) of arterial stiffness among healthy subjects (n = 24)

**Pulse Wave Velocity**	**Observer I**	**Observer II**	**Pearson (r)**	**95% CI**	**P value**
Heart Brachial PWV	262.4 ± 16.09	262.4 ± 18.28	0.88	0.75 to 0.95	p < 0.0001
Heart Ankle PWV	425.4 ± 17.96	424.9 ± 16.96	0.91	0.80 to 0.96	p < 0.0001
Brachial Ankle PWV	1124 ± 104	1106 ± 105.2	0.89	0.76 to 0.95	p < 0.0001
Carotid Femoral PWV	706.2 ± 90.36	699.2 ± 92.53	0.90	0.79 to 0.96	p < 0.0001
Brachial ASI	37.80 ± 3.93	37.01 ± 4.83	0.62	0.28 to 0.82	p < 0.001
Ankle ASI	48.39 ± 8.54	48.06 ± 6.71	0.85	0.69 to 0.94	p < 0.0001

Values are expressed as Mean ± SD

**Table 4:** Means and correlational analysis (Pearson r value) of arterial stiffness among CAD Patients (n = 20)

**Pulse Wave Velocity**	**Observer I**	**Observer II**	**Pearson (r)**	**95% CI**	**P value**
Heart Brachial PWV	298.9 ± 59.26	298.9 ± 59.61	0.99	0.97 to 0.99	p < 0.0001
Heart Ankle PWV	550.3 ± 83.60	553.5 ± 87.53	0.98	0.96 to 0.99	p < 0.0001
Brachial Ankle PWV	1719 ± 368.8	1731 ± 364.8	0.99	0.98 to 0.99	p < 0.0001
Carotid Femoral PWV	1199 ± 307.4	1210 ± 304	0.98	0.97 to 0.99	p < 0.0001
Brachial ASI	39.15 ± 14.21	38.63 ± 15.07	0.93	0.85 to 0.97	p < 0.0001
Ankle ASI	60.48 ± 17.77	60.70 ± 16.09	0.95	0.88 to 0.98	p < 0.0001

Values are expressed as Mean ± SD

**Table 5:** Means and correlational analysis (Pearson r value) of arterial stiffness among all subjects (Healthy + CAD) (n = 44)

**Pulse Wave Velocity**	**Observer I**	**Observer II**	**Pearson (r)**	**95% CI**	**P value**
Heart Brachial PWV	279.0 ± 45.05	279.0 ± 45.67	0.98	0.96 to 0.99	p < 0.0001
Heart Ankle PWV	482.2 ± 84.97	483.3 ± 87.94	0.99	0.98 to 0.99	p < 0.0001
Brachial Ankle PWV	1394 ± 394.7	1390 ± 404.5	0.99	0.98 to 0.99	p < 0.0001
Carotid Femoral PWV	940.7 ± 331.7	941 ± 338.3	0.99	0.98 to 0.99	p < 0.0001
Brachial ASI	38.41 ± 9.8	37.74 ± 10.65	0.91	0.85 to 0.95	p < 0.0001
Ankle ASI	53.88 ± 14.69	53.81 ± 13.38	0.94	0.90 to 0.96	p < 0.0001

Values are expressed as Mean ± SD

In the control group, the Pearson's correlation coefficient was 0.88, 0.91, 0.89 and 0.90 (p < 0.0001) for hb, ha, ba and C-F PWV respectively. The coefficient was 0.85 for ankle ASI with p < 0.0001. Similarly in CAD group, and in data from all subjects combined also the correlation was highly significant, suggesting high reproducibility of our method for PWV determination.

**Figure 4:**
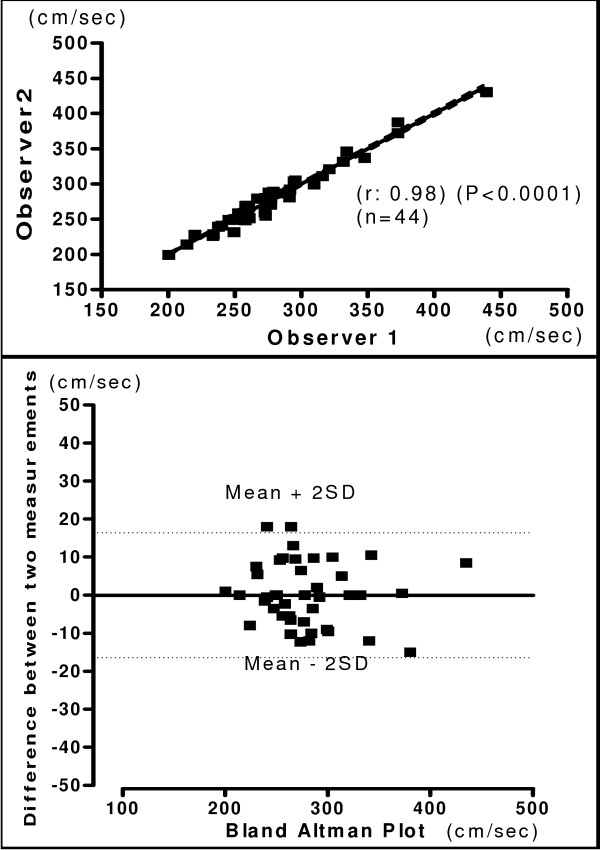
**Interobserver Heart Brachial PWV**. Relationship between two independent measurements of hb PWV by two observers (Upper panel). Reproducibility of hb PWV. Bland-Altman plot showing observer difference in measurements. (Lower panel).

**Figure 5:**
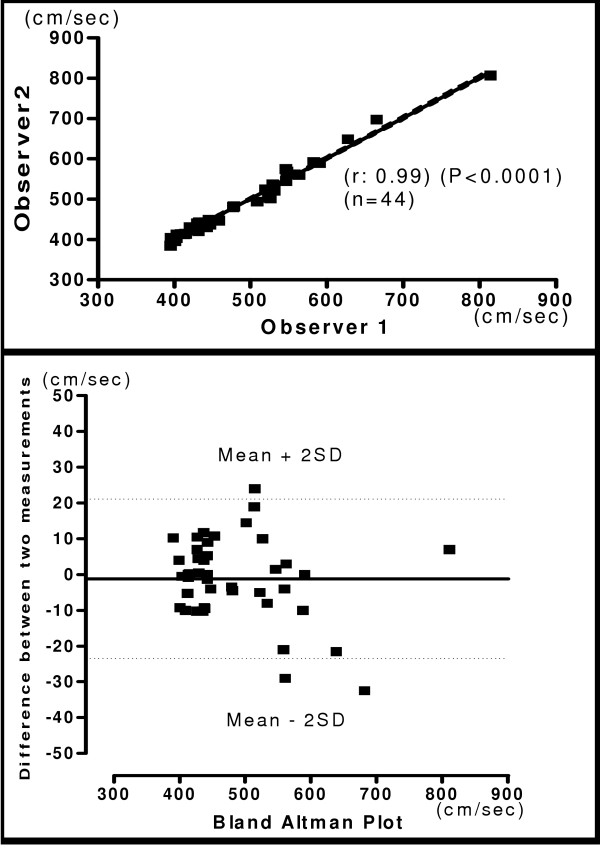
**Interobserver Heart Ankle PWV**. Relationship between two independent measurements of ha PWV by two observers (Upper panel). Reproducibility of ha PWV. Bland-Altman plot showing observer difference in measurements. (Lower panel).

**Figure 6:**
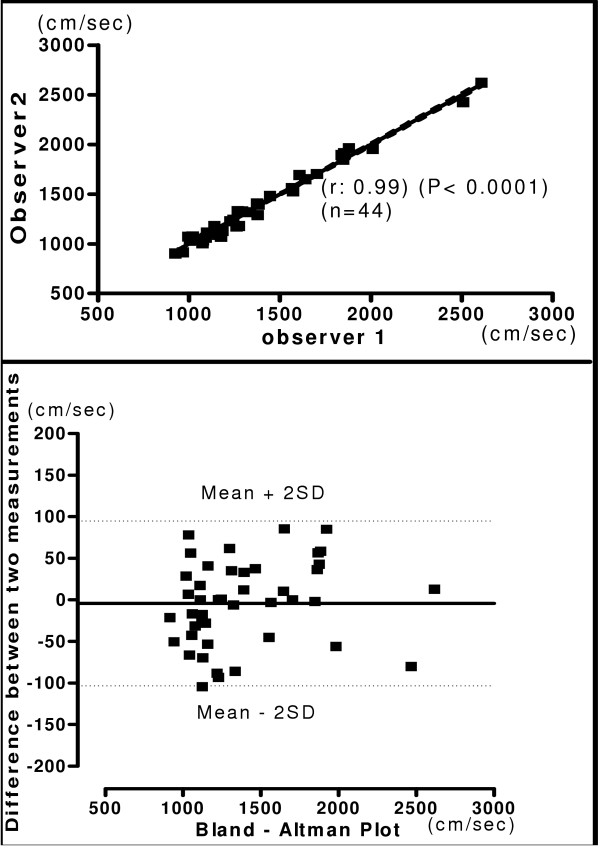
**Interobserver Brachial Ankle PWV**. Relationship between two independent measurements of ba PWV by two observers (Upper panel). Reproducibility of ba PWV. Bland-Altman plot showing observer difference in measurements. (Lower panel).

**Figure 7:**
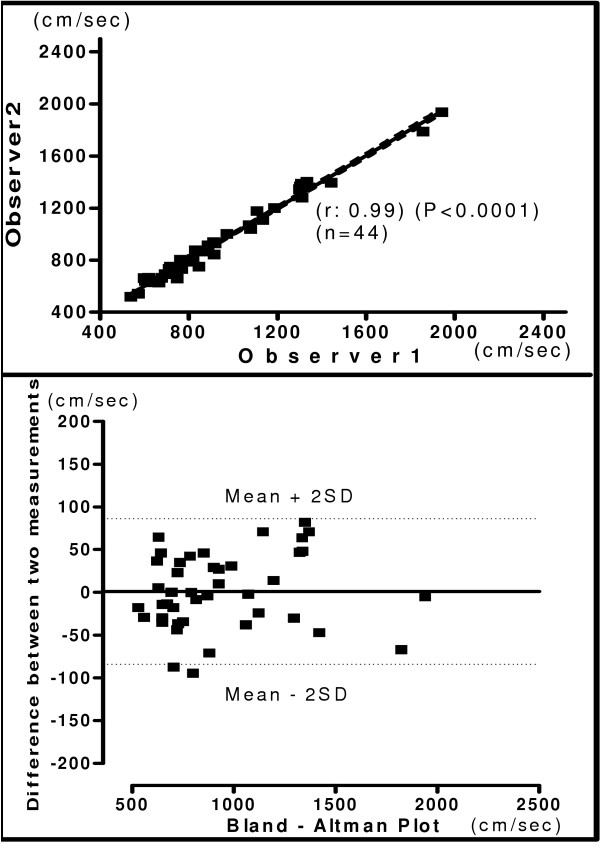
**Interobserver Carotid Femoral PWV**. Relationship between two independent measurements of C-F PWV by two observers (Upper panel). Reproducibility of C-F PWV. Bland-Altman plot showing observer difference in measurements. (Lower panel).

The relationship and Bland-Altman plot for hb, ha, ba and C-F PWV obtained by two observers is shown in figures 4, 5, 6 and 7 respectively. A very good correlation was observed between the two measurements (r = 0.98 – 0.99) in all these above parameters. There was no trend for the reproducibility of measurements to vary with the underlying mean values in any of the parameters analyzed. In the Bland-Altman Plots of interobserver measurements of PWV, there was no significant difference in the values for reproducibility reported between observers and most of the values ranged within a mean ± 2 SD.

**Figure 8:**
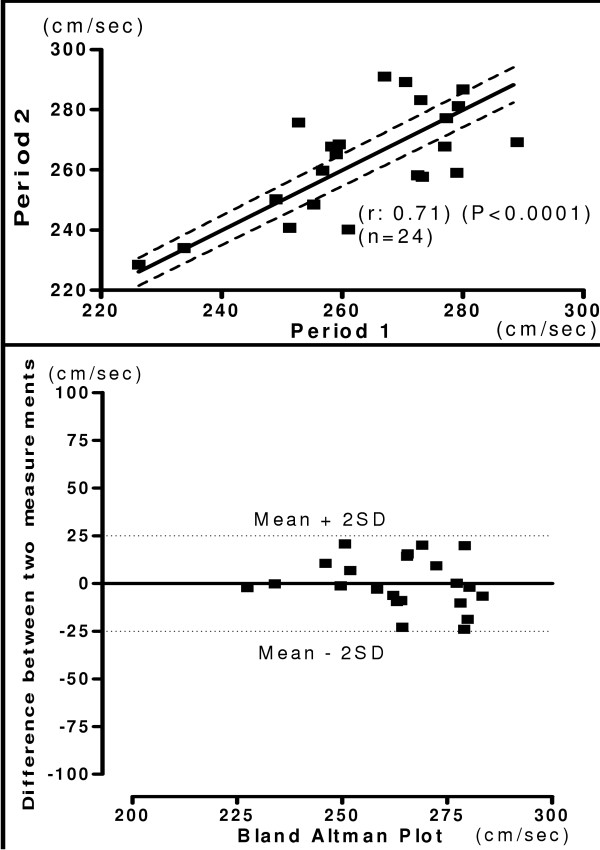
**Interperiod Heart Brachial PWV**. Relationship between measurements of hb PWV between two different periods (Upper panel). Reproducibility of hb PWV. Bland-Altman plot showing difference in measurement between two periods. (Lower panel).

**Figure 9:**
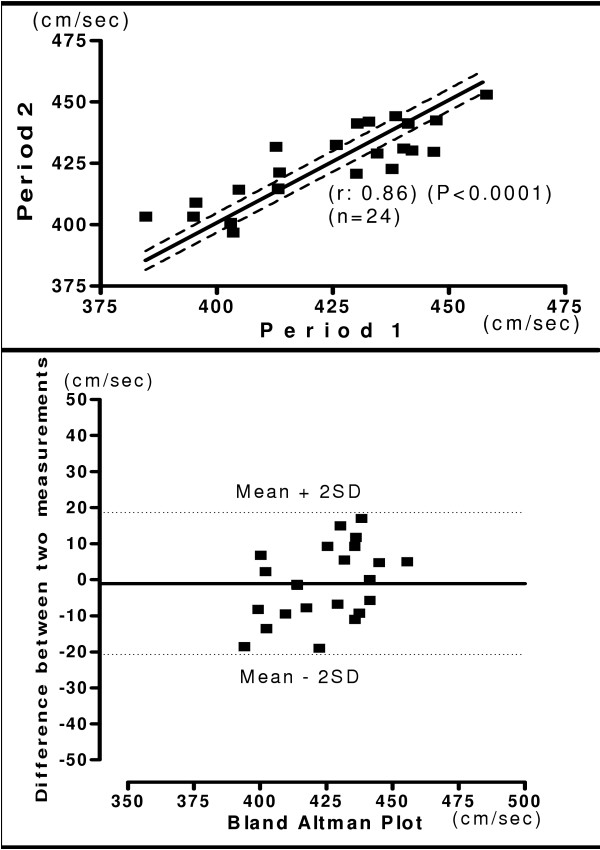
**Interperiod Heart Ankle PWV**. Relationship between measurements of ha PWV between two different periods (Upper panel). Reproducibility of ha PWV. Bland-Altman plot showing difference in measurement between two periods. (Lower panel).

**Figure 10:**
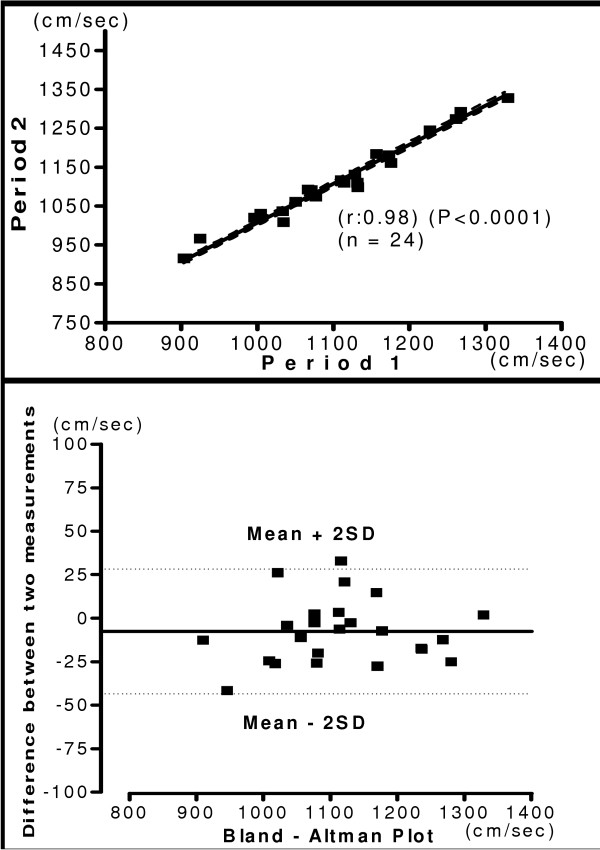
**Interperiod Brachial Ankle PWV**. Relationship between measurements of ba PWV between two different periods (Upper panel). Reproducibility of ba PWV. Bland-Altman plot showing difference in measurement between two periods. (Lower panel).

**Figure 11:**
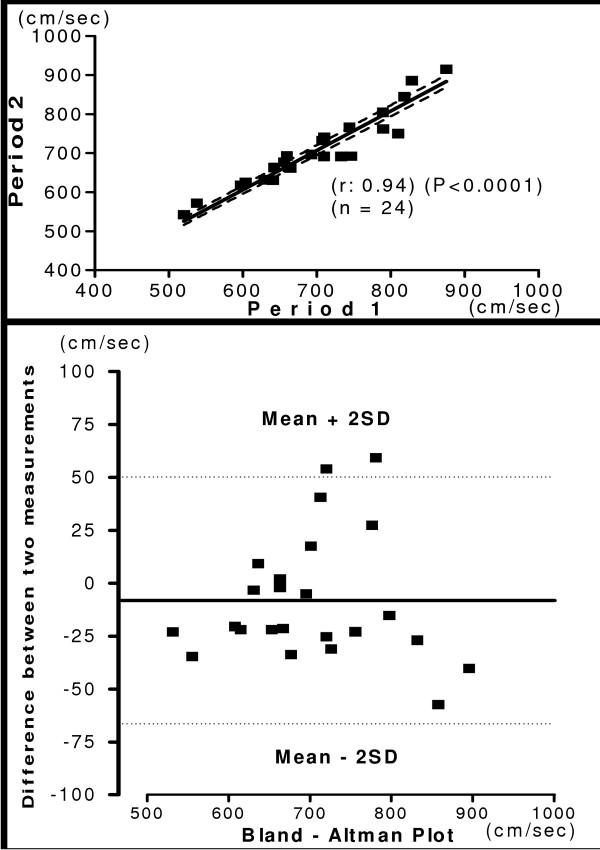
**Interperiod Carotid Femoral PWV**. Relationship between measurements of C-F PWV between two different periods (Upper panel). Reproducibility of C-F PWV. Bland-Altman plot showing difference in measurement between two periods. (Lower panel).

The mean of hb, ha, ba, C-F PWV and ASI recorded during two-separate occasions in same subject was shown in the table 6. The relationship and Bland-Altman plot of two period measurements are shown in figure 8, 9, 10 and 11. The measurements of PWV had significant correlation with (r = 0.71 – 0.98). There was significantly less variation in reproducibility as clearly shown in Bland-Altman plot. Most of the values of PWV are lying within the mean ± 2 SD. The present study demonstrated, considerably high Pearson's correlation coefficients for both interobserver and interperiod reproducibility in PWV measurements with new device. Correlation analysis of carotid femoral PWV with other PWV parameters obtained is shown in table 7. Both brachial and ankle ASI, hb, ha, and ba PWV correlated significantly with C-F PWV. The Pearson's correlation coefficient was 0.99 for brachial ankle PWV and was 0.30 for brachial ASI.

**Table 6:** Means and correlational analysis (Pearson r value) of arterial stiffness among all subjects (Healthy + CAD) (n = 24)

**Pulse Wave Velocity**	**Period I**	**Period II**	**Pearson (r)**	**95% CI**	**P value**
Heart Brachial PWV	263.6 ± 15.38	263.6 ± 17.59	0.71	0.43 to 0.86	p < 0.0001
Heart Ankle PWV	424.4 ± 19.73	425.5 ± 15.74	0.86	0.70 to 0.93	p < 0.0001
Brachial Ankle PWV	1111 ± 105.7	1119 ± 104.1	0.98	0.96 to 0.99	p < 0.0001
Carotid Femoral PWV	698.6 ± 91.28	706.8 ± 91.58	0.94	0.96 to 0.97	p < 0.0001
Brachial ASI	37.08 ± 4.72	37.27 ± 3.95	0.84	0.67 to 0.93	p < 0.0001
Ankle ASI	48.88 ± 7.10	46.99 ± 7.85	0.81	0.65 to 0.91	p < 0.0001

Values are expressed as Mean ± SD

**Table 7:** The correlation between carotid-femoral PWV and other variables evaluated in all subjects (Healthy + CAD) (n = 88)

	P-Value	Pearson (r)
Brachial ASI	<0.005	0.30
Ankle ASI	<0.0001	0.67
Heart Brachial PWV	<0.0004	0.37
Heart Ankle PWV	<0.0001	0.83
Brachial Ankle PWV	<0.0001	0.99

ASI, arterial stiffness index; PWV, pulse wave velocity

Our present study was not aimed to detect differences in the measured parameters between control group and CAD group; therefore no sub group analysis was performed. The CAD group was included to provide a wide range of PWV values, thus strengthening the reproducibility of method.

## Discussion

In recent years with the development of readily available noninvasive assessment techniques investigation of arterial stiffness, especially of the large arteries has gathered pace. These include the measurement of PWV, the use of ultrasound to relate the change in diameter or area of an artery to distending pressure and analysis of arterial wave forms obtained by applanation tonometry. For the measurement of AS, several new techniques have been developed, but their association with aortic PWV, an established measure of central arterial stiffness has not been validated. Because of its size and elasticity, the aorta is the main determinant of systemic arterial compliance [16] and thus AS models incorporating wave reflection are influenced by both central and peripheral AS. Central stiffness is most commonly assessed using aortic PWV, which is a robust measurement and predictive of cardiovascular morbidity in hypertensive [17] and non-hypertensive subjects [18,19]. Aortic PWV can also be measured non-invasively by using MRI [20]. It has potential advantage of accurate determination of path length, however its use is very much limited due to prolonged time required to make recording, lack of availability in the clinical settings, high cost for measurement and difficulty in performing within a strong magnetic field.

Perhaps the best and most widely used technique to estimate the distensibility and stiffness of the aorta and proximal vessels is PWV. Although the properties of large arteries have been studied for several decades, the field of clinical arterial biomechanics remains in its infancy. The clinical value of any of the available techniques is yet to be proved convincingly and no single parameter of compliance or stiffness can ever be expected to describe all clinically relevant arterial wall properties. Measurement error can be substantial, including problems related to the measurement of both transit time and distance traveled by the pulse wave. There is a need for a simple, reliable, noninvasive method of detecting early disturbances at the time when therapeutic intervention can be most beneficial. Currently, none of the methodologies available are yet suitable for use in wide spread clinical practice. The results of our study indicate that a new device provides a simple non-invasive method for the assessment of arterial stiffness and PWV in clinical settings and operator does not require prolong period of training to achieve measurements.

We have determined the C-F PWV along with determination of other velocities like hb, ha, ba PWV. The present experiment included both healthy control and patients with CAD to provide a wide range of values. The correlation with C-F PWV was very high with other PWV estimated. Analysis of data was done by using Bland-Altman Plots and reproducibility was reported as the SD of the differences between the paired measurements. This method was used rather than only reporting coefficient of variance, as the latter is a less satisfactory method of assessing reproducibility and can some time misleading [21].

Arterial stiffness index (ASI) also well correlated with C-F PWV (r = 0.67, p < 0.0001). Similar correlation between ASI and C-F PWV has been documented earlier in elderly hypertensive patients [22]. Our results indicate that, non-invasive PWV measurements with new device were reproducible. Both within observer and between the observers, reproducibility was high with low SD for measurement differences. The lower value for between observer differences reflects the fact that, the means of two readings made by each observer are comparable, thus reducing variability. The method we used to measure PWV does not require any specialized technique and the examiner only has to wrap cuffs on all the limbs and place electrodes for recording lead II ECG. Our study demonstrated high Pearson's correlation coefficient for interobserver and interperiod reproducibility. In the Bland-Altman plot the deviation was greater at high PWV values. A similar deviation has also been reported in the measurements of C-F PWV [23]. It is known that, the variability in measurement of PWV increases when PWV are high due to confounding factors like high blood pressure, blood flow [24], and high sympathetic tone [25]. In our study though we have included CAD patients, the deviation in measurements ranged within a mean ± 2SD even with high PWV. Furthermore both interobserver and interperiod coefficient variations were less than 20 %. We made comparison of interobserver and interperiod measurements with both Bland-Altman and correlation analysis. Unlike correlation analysis, Bland-Altman Plots do not assume zero error for either of the two measurements under comparison and therefore is a better indicator of the true agreement between the measurements [26]. We noted significant association of correlations analysis between C-F PWV and other PWV and ASI, with low variability in Bland-Altman analysis.

There are a number of different ways to measure PWV, and these are generally simple to perform.

## Conclusion

The results of this study indicate that, the measurement of arterial PWV by oscillometric technique using PeriScope is reproducible and simple. The validity and reproducibility of the measurement of PWV in interobserver and interperiod evaluation was high with a low SD for measurement difference. Derived carotid femoral velocity correlated well with hb, ha and ba PWV and arterial stiffness index. Simultaneous measurement of all velocities and stiffness index provide better over all assessment of arterial functions. New device may be useful in studying arterial stiffness in large population.

## List of Abbreviations

**ABI **– Ankle Brachial Index

**AS **– Arterial Stiffness

**ASI **– Arterial Stiffness Index

**ba **– Brachial ankle

**BP **– Blood Pressure

**CAD **– Coronary Artery Disease

**C-F **– Carotid Femoral

**CV **– Coefficient of variation

**CVD **– Cardiovascular Disease

**DBP **– Diastolic blood pressure

**DSP **– Digital signal processing

**ECG **– Electrocardiogram

**ha **– Heart ankle

**hb **– Heart brachial

**HR **– Heart rate

**MAP **– Mean Arterial Pressure

**OPD **– Out Patient Department

**PP **– Pulse pressure

**PTT **– Pulse Transit Time

**PWV **– Pulse Wave Velocity

**SBP **– Systolic blood pressure

**SD **– Standard Deviation

**SE **– Standard Error

**UT **– Upstroke Time

## Authors' contributions

MURN conceived and designed the study and drafted the manuscript. BMR and YS performed the study as two separate observers under the supervision of MURN. YS compiled and analyzed the data statistically. ANP performed and evaluated the angiogram of cardiac patients. PUR participated in the design and coordination of the study. All authors read and approved the final manuscript.
